# Soybean Cyst Nematode Population Development and its Effect on Pennycress in a Greenhouse Study

**DOI:** 10.2478/jofnem-2022-0006

**Published:** 2022-04-11

**Authors:** Cody Hoerning, Senyu Chen, Katherine Frels, Donald Wyse, Samantha Wells, James Anderson

**Affiliations:** 1 Department of Agronomy and Plant Genetics, University of Minnesota, St. Paul, MN 55108; 2 Department of Plant Pathology, University of Minnesota, St. Paul, MN 55108

**Keywords:** alternative host, *Heterodera glycines*, host–parasitic relationship, host suitability, pennycress, *Thalapsi Arvense L.*, soybean cyst nematode

## Abstract

Midwest crop production is dominated by two summer annual crops grown in rotation, viz., corn (*Zea mays* L.) and soybean (*Glycine max* L.). Winter oilseed crops, such as pennycress (*Thlaspi arvense* L.), can provide ecosystem and economic benefits when added to the corn–soybean rotation. However, adding a new crop adds risks, such as increased pest pressure. The objectives of this study were to (i) evaluate population development of three soybean cyst nematode (SCN; *Heterodera glycines*) biotypes on three pennycress genotypes and susceptible soybean and (ii) determine whether SCN inoculation level influenced plant biomass. SCN population density and biomass were determined after 60 d in the greenhouse. At the inoculation level of 2,000 eggs/100 cm^3^ soil, the average egg density for the three pennycress genotypes was 1,959 eggs/100 cm^3^ soil, lower than that for the susceptible soybean ‘Sturdy’ (9,601 eggs/100 cm^3^ soil). At the inoculation level of 20,000 eggs/100 cm^3^ soil, the average egg density for the three pennycress genotypes was 6,668 eggs/100 cm^3^ soil, lower than that for ‘Sturdy’ (40,740 eggs/100 cm^3^ soil). The inoculation level did not affect plant biomass. Pennycress is an alternative host to SCN under greenhouse conditions but is a less suitable host than soybean.

Midwest agriculture in the United States is dominated by two crop species, viz., corn (*Zea mays* L.) and soybean (*Glycine max* [L.] Merr.). Corn and soybean are commonly rotated annually, and together, they account for 39.8 million hectares harvested in the eight-state Midwest region of the United States (*United States Department of Agriculture* [USDA]- National Agricultural Statistics Service, 2022). As summer-annual crops, corn and soybean are planted in April–May and harvested in October–November. One-half of the total annual precipitation falls in the Midwest during the spring and autumn months (Midwestern Regional Climate Center [MRCC], 2020). During this period, the lack of vegetative cover results in nutrient loss from the system and the deposition of nitrates in groundwater. Many Midwestern watersheds have vulnerable aquifers and high nitrogen loading from agriculture, resulting in high nitrate levels in drinking and recreational water sources (Nolan and Hitt, 2006; Van Metre *et al*., 2016).

Pennycress (*Thlaspi arvense* L.) is a harvestable winter annual oilseed crop intended for inclusion in Midwest corn and soybean rotations. Enhancing production and providing ecosystem services, such as reducing nitrates in the soil, comprise the primary focus of incorporating pennycress into these rotations (Heaton *et al*., 2013). Preliminary research indicates that adding winter oilseeds to summer-annual soybean rotation can produce a total seed oil yield (winter oilseeds + soybean) up to 50% greater than the monocultured soybeans (Gesch *et al*., 2014; Dose *et al*., 2017).

Potential challenges are associated with adding a recently domesticated crop to a rotation. One identified challenge is increased pest pressure (Chen *et al*., 2018). Pests that have multiple crop hosts are of particular concern. In addition, adding an alternative host into a crop rotation may temporally extend the pest life cycle. Extending pest life cycles to alternative hosts in a growing season is known as the “green bridge effect” and can result in crop damage and lower yields in rotation (Belder *et al*., 2008; Mahoney *et al*., 2016).

The soybean cyst nematode (SCN; *Heterodera glycines*) is the most prevalent pest affecting soybean. Soybean yield losses caused by SCN are estimated at an average of 3.1 million metric tons annually in the United States (Bradley *et al*., 2021). Soybean growers protect soybean yields through multiple practices aimed at managing SCN, including the use of resistant soybean cultivars, crop rotation, and use of nematicidal crop protection products (Chen *et al*., 2001; Hassan *et al*., 2013; McCarville *et al*., 2017). Agronomic crops that are known alternative hosts to SCN include beans (such as mung, dry, and snap beans), clover, pea, lupine, and vetch species (Riggs and Wrather, 1992; Smith and Young, 2003). Growers managing SCN are advised to avoid these alternative host crops in rotation when SCN numbers in a field are >5,000 eggs/100 cm^3^ soil (Tylka, 2019).

Some weeds have also been identified as alternative hosts to SCN. Managing alternative host weeds in rotation is another management tool recommended for SCN. Numerous weed species, 116 in total, have been identified as alternative hosts to SCN. The major plant families included in this count are Fabaceae with 58 species, Brassicaceae with nine species, and Asteraceae with eight species (Rocha *et al*., 2021). Chickweed, henbit, purple deadnettle, purslane, shepherd’s purse, and wild mustard have been identified as alternative weed hosts in Midwest agricultural production systems (Creech *et al*., 2008; Johnson *et al*., 2008; Basnet *et al*., 2019). Pennycress (family: Brassicaceae), the crop intended for oilseed–soybean rotation, was also reported to be an alternative host in these weed surveys. Weed surveys classify pennycress as a low-to-moderate alternative host of SCN, representing 27%–34% of the population density observed on the susceptible soybean control (Venkatesh *et al*., 2000; Poromarto and Nelson, 2010; Poromarto *et al*., 2015). These weed survey experiments were limited in identifying host status without considering the SCN biotype-and-host genotype interactions. In addition, the pennycress genotypes tested in these surveys were often wild collections taken from a field and, therefore, had variation in the genetic background of the plants tested. Before implementing pennycress into a corn–soybean rotation, a better understanding of the host–pathogen interaction is necessary. The objectives of this study were as follows: (i) evaluate population development of three SCN biotypes on three pennycress genotypes and a susceptible soybean; and (ii) determine whether SCN inoculation level influenced plant biomass production.

## Materials and Methods

A greenhouse experiment was conducted in the year 2016 and repeated in 2018 to evaluate the development of SCN population on pennycress. SCN-free soil was collected from a field at the University of Minnesota Southern Research and Outreach Center in Waseca, *Minnesota*. The soil was a Nicollet clay loam (fine–loamy, mixed, superactive, mesic Aquic Hapludoll) with a pH of 6.1 and an organic matter content of 3.8%. The particle size distribution was 40% sand, 35% silt, and 25% clay. The soil was screened, mixed, distributed to four lots, and heat-treated at 48°C in a commercial oven for 24 hr. This temperature is routinely used to keep alive the beneficial soil microbes required for plant growth and kill the SCN eggs present in soil and cysts (Hu *et al*., 2016). The soil was mixed again after heating and divided into 1-kg lots. A small quantity of sand (500 g) was added to each lot to facilitate drainage.

SCN populations classified as HG Types 0, 1.3.6, and 2.5.7 were collected from fields in, respectively, Swift, Murray, and Waseca counties, Minnesota. These populations, hereafter referred to as SCN biotypes, were selected to represent different types of virulence to commonly used sources of resistance in soybean, namely, PI88788 and Peking. The female index (FI) was calculated by comparing the number of SCN females present on the indicator line divided by the number of females present on a susceptible soybean variety multiplied by 100. Resistance was defined as an FI value <10 compared to a susceptible soybean variety (Niblack *et al*., 2002). In the HG [*Heterodera glycines*] Type classification, the Peking indicator line is Line 1, and the PI88788 indicator line is Line 2. The seven soybean plant indicator lines described by Niblack *et al*. (2002) were used for the HG type test. The three biotypes chosen had FI values >10 on Peking (HG Type: 1.3.6), >10 on PI88788 (HG Type: 2.5.7), and <10 on both Peking and PI88788 (HG Type: 0). The collected biotypes were cultured on SCN-susceptible soybean cv. Sturdy in autoclaved soil inside a growth room at 28°C. The autoclaved soil was treated at 121°C and 110 kPa for 180 min. SCN females and cysts were collected from the roots of 45-d-old soybean plants to ensure uniform ages of the SCN eggs among the three biotypes. Newly formed females and cysts were washed off soybean roots with a vigorous water stream through an 850-μm-aperture sieve onto a 250-μm-aperture sieve and extracted by centrifugation in 76% (w/v) sucrose solution (Liu and Chen, 2000). The eggs were released by crushing cysts on a 150-μm-aperture sieve with a rubber stopper mounted on a motor and collected on a 25-μm-aperture sieve (Faghihi and Ferris, 2000). The eggs were separated from debris by centrifugation in a 35% (w/v) sucrose solution for 5 min at 1,500 g (Liu and Chen, 2000). The eggs were then transferred to an antibiotic solution (streptomycin sulfate, chlortetracycline, and 8-quinolinol at 100 mg/l, 50 mg/l, and 20 mg/ll, respectively) and maintained at 4°C before being used within 1 d. The eggs (2,000 or 20,000 eggs/100 cm^3^ soil) were diluted in 10 ml of water and mixed into each lot of soil. The egg-and-soil mixture was placed into a 16-cm-diameter plastic pot.

Pennycress seedlings were germinated in 106 cm^3^ cone-tainers (Ray Leach SC7U, Stuewe & Sons, Inc., Tangent, Oregon), with one plant per cone-tainer. After 21 d, the seedlings were moved to a growth chamber set to 12-h light/d and 4°C for a 21-d vernalization treatment. Next, the plants were transplanted to pots containing the SCN-infested soil. Four plants were transplanted to each pot. For the soybean treatments, 10 soybean seeds were planted per pot. After emergence, the soybean seedlings were thinned to four plants per pot. The pots were maintained in the greenhouse with the temperature set at 28°C and daylight of 16 h. The pots were watered daily to water-holding capacity. Fertilizer was applied every 2 wk at a rate of 25 ml of Peters Hydrop-Sol 5-11-26 (Carlin Horticultural Supplies, St. Paul, Minnesota) per pot. Sixty days after inoculation, the experiment was terminated. The shoots were cut at the soil level, and the samples were dried for 3 d at 40°C to obtain dry matter. The samples were weighed, and the weight of the dry shoot biomass (in grams) was determined.

The soil in each pot was thoroughly mixed, the roots removed, and a soil subsample of 100 cm^3^ was used to extract cysts using a semiautomatic elutriator (Byrd *et al*., 1976). The females formed on the roots were washed vigorously with a water stream through an 800-μm-aperture sieve onto a 250-μm-aperture sieve. The cysts from the roots and elutriation process were combined and separated from debris by centrifugation in 76% (w/v) sucrose solution. The cysts were poured from the centrifuge tube directly onto a 150-μm-aperture sieve. The eggs were released by crushing the cysts on a 150-μm-aperture sieve with a rubber stopper mounted on a motor and collected on a 25-μm-aperture sieve (Faghihi and Ferris, 2000). An aliquot (1 ml) of egg suspension was used to count the eggs. The number of eggs per 100 cm^3^ of soil was determined.

The experimental design was a 4 × 3 × 3 factorial, arranged in a randomized complete block design with four replicates. The first factor consisted of three genotypes of pennycress from the University of Minnesota (UMN) pennycress breeding program (PC103, PC106, and PC108) and a susceptible soybean cultivar (‘Sturdy’) from the UMN soybean breeding program. The second factor, SCN biotype, consisted of three levels: HG Types 0, 1.3.6, and 2.5.7. The third factor, inoculation, had three levels. The inoculation levels used were 0, 2,000, and 20,000 eggs/100 cm^3^ soil. Each replication was placed on a separate greenhouse bench (block). The experimental unit was an individual pot containing the plants. The SCN reproductive factor (RF) of the experiment was determined by dividing the final SCN population density (P_final_ = egg density at experiment termination) by the initial SCN population density (P_initial_ = egg density at inoculation).

### Statistical analysis

Data were analyzed using linear mixed-effect models in R (R Core Team, 2020). The experiment was a factorial design, with each treatment occurring independently. Analytical assumptions for the mixed analysis of variance were examined by graphical inspection of the residual plots. If the assumptions of equal variance or normality were not met, a generalized linear mixed-effect model with a negative binomial or Poisson distribution error structure was used (packages lme4, MASS [Modern Applied Statistics with S]; Venables and Ripley 2002; Bates *et al*. 2015). The two experiments conducted in 2016 and 2018 were independent and revealed homogeneous variances. Therefore, the data for the two experiments were combined and analyzed together. Block and year were treated as random effects in all models. Crop (C), SCN biotype (B), and inoculation (I) were treated as fixed effects. Likelihood-ratio χ^2^-test was used to assess the significance of the main effects and their interactions (Fox and Weisberg 2019). Mean separation was done using Tukey–Kramer least-square means with an associated *P* < 0.05. Two-sided *t*-tests were performed on the crops to determine whether the final egg density was different (*P* < 0.05) from the initial inoculation egg density. The zero-inoculation level (control) treatment caused model convergence issues because of multiple zeroes for SCN population density and RF; as such, the control treatment was omitted in these analyses. For the figure and the tables, means are reported on the original scale to ease discussion.

## Results

### SCN egg population densities

**Figure 1:**
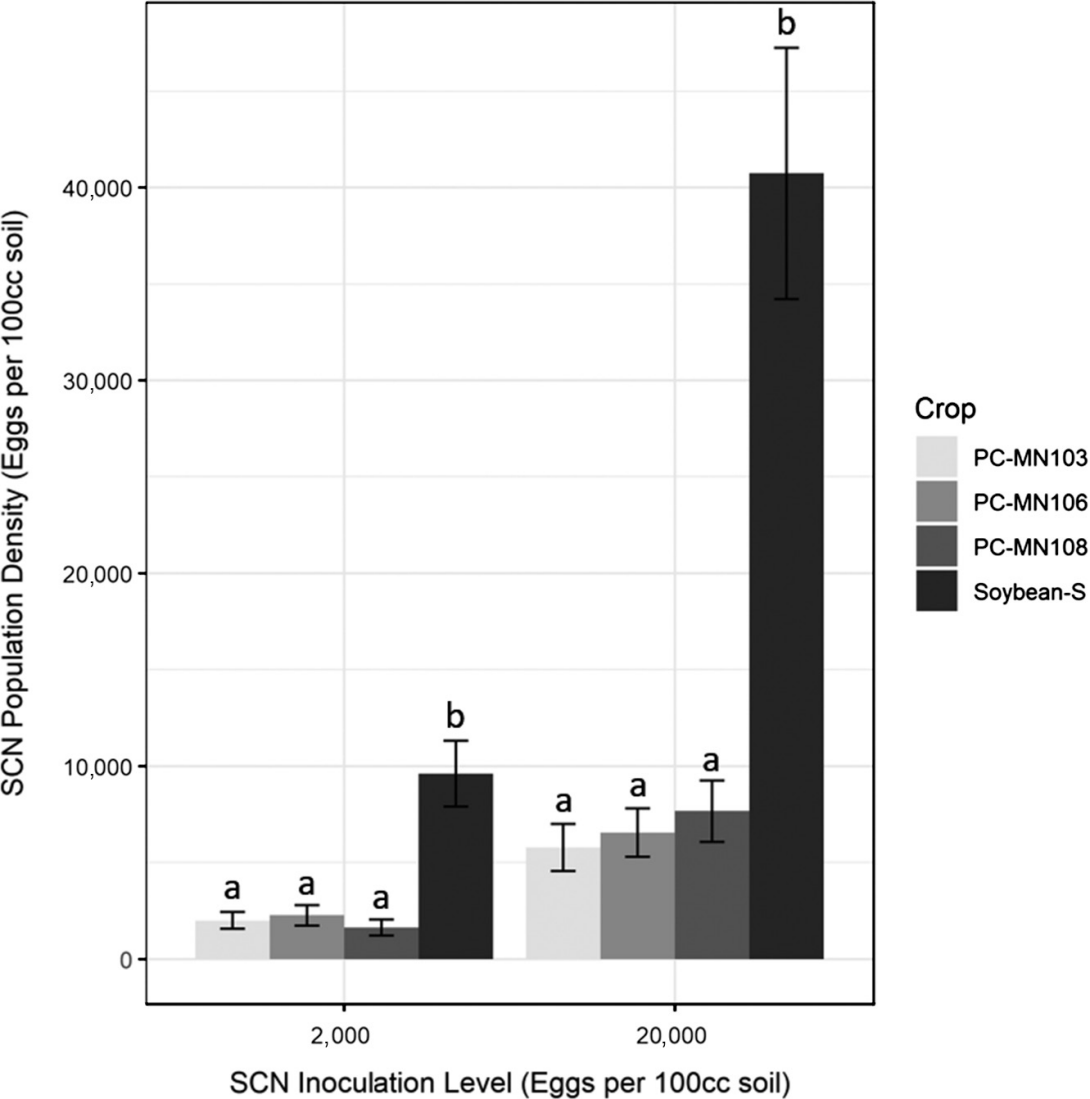
Influence of inoculation level and crop treatment on final SCN egg population density in the greenhouse evaluation experiment. Soybean-S was soybean genotype ‘Sturdy’; PC-MN103 was pennycress genotype ‘MN103’; PC-MN106 was pennycress genotype ‘MN106’; and PC-MN108 was pennycress genotype ‘MN108’. The genotypes were sourced from the University of Minnesota pennycress and soybean breeding programs. Error bars denote standard error. Within an inoculation level, bars with the same lowercase letter did not differ in SCN population density using Tukey–Kramer least-square means (*P* < 0.05). SCN, soybean cyst nematode.

Likelihood-ratio χ^2^-test revealed that the main factors crop (C) and inoculation (I) were significant (*P* < 0.05). The interaction of crop and inoculation (C × I) was also significant (Table 1). The interaction C × I was investigated (Fig. 1). In Figure 1, the two inoculation levels, 2,000 and 20,000 eggs/100 cm^3^ soil, were separated to analyze the within-group differences of crop treatment. When within-inoculation means were examined, the SCN egg density on pennycress represented 16% (inoculation level of 20,000 eggs/100 cm^3^ soil) and 20% (inoculation level of 2,000 eggs/100 cm^3^ soil) of the density on the susceptible soybean ‘Sturdy’. The *t*-tests (test value = 2,000) of the individual crops showed that at the inoculation level of 2,000 eggs/100 cm^3^ soil, there was no significant difference (*P* < 0.05) between the initial inoculation level on the three pennycress genotypes and the final population density. The *t*-test (test value = 2,000) of soybean showed a significant difference from the initial inoculation level, as the final population density increased 380% (Table 2). For the *t*-tests (test value = 20,000) of the individual crops at the inoculation level of 20,000 eggs/100 cm^3^ soil, there was a significant difference (*P* < 0.05) between the initial inoculation level and the final population density on all four crops. The percentage decrease ranged from 62% to 71% on the three pennycress genotypes. For soybean at the inoculation level of 20,000 eggs/100 cm^3^ soil, the final population density increased 104% over the initial inoculation level (Table 3).

**Table 1:** Likelihood-ratio χ^2^-test for SCN population density, as influenced by crop ([C]: MN103, MN106, MN108, and soybean), HG type (HG Types 0, 2.5.7, and 1.3.6), and inoculation ([I]: 0, 2,000, and 20,000 eggs/100 cm^3^ soil).

**Source of fixed variation**	***χ^2^***	**Numerator Df**	***P*-value**
Crop (C)	96.1514	3	< 2.2e−16^***^
HG type (H)	1.4823	2	0.4766
Inoculation (I)	38.4078	1	5.74e−10^***^
C × H	2.9414	6	0.8162
C × I	38.9127	3	1.81e−08^***^
H × I	1.1895	2	0.5517
C × H × I	5.5155	6	0.4796

*** represents significance of the χ^2^-test at the 0.001 level.

Df = degrees of freedom; HG, *Heterodera glycines*; SCN, soybean cyst nematode.

**Table 2:** Two-sided *t*-test results of the crop treatments at inoculation level of 2,000 eggs/100 cm^3^ soil.

**Crop**	**Mean**	***t***	**Df**	**Sig. *t***
MN 103	1,999	−0.003	22	0.999
MN 106	2,258	0.493	23	0.627
MN 108	1,621	−0.908	22	0.374
Soybean	9,601	4.437	23	0.0002^***^

The test value was 2,000; *t* = *t*-statistic; Df = degrees of freedom.

*** represents the significance of the two-sided *t*-test at the 0.001 level.

**Table 3:** Two-sided *t*-test results of the crop treatments at inoculation level of 20,000 eggs/100 cm^3^ soil.

**Crop**	**Mean**	***t***	**Df**	**Sig. *t***
MN 103	5,777	−11.712	20	2.09e−10^***^
MN 106	6,559	−10.676	23	2.19e−10^***^
MN 108	7,667	−7.738	23	7.55e−08^***^
Soybean	4,0739	3.185	23	0.004^**^

The test value was 20,000. *t* = *t*-statistic, Df = degrees of freedom.

** and ^***^represent significance of two-sided *t*-test at 0.01 and 0.001, respectively.

### Tests for SCN RF

Likelihood-ratio χ^2^-test for SCN RF revealed that the main factors crop (C) and inoculation (I) were significant (*P* < 0.05) and the interaction of crop and inoculation (C × I) was also significant (Table 4). The interaction C × I was investigated (Table 5). When within-inoculation means were examined, there was no significant difference between the three pennycress genotypes tested at both inoculation levels. However, the three pennycress genotypes were significantly different (*P* < 0.05) from the susceptible soybean at both levels. The RF on pennycress genotypes was on average 0.98 vs 4.80 for susceptible soybean at the inoculation level of 2,000 eggs/100 cm^3^ soil. At the inoculation level of 20,000 eggs/100 cm^3^ soil, the RF for the pennycress genotypes averaged 0.33, and the susceptible soybean RF was 2.04.

**Table 4:** Likelihood-ratio χ^2^-test for RF, as influenced by crop ([C]: MN103, MN106, MN108, and soybean), HG type (HG Types 0, 2.5.7, and 1.3.6), and inoculation ([I]: 0, 2,000, and 20,000 eggs/100 cm^3^ soil).

**Source of fixed variation**	**χ^2^**	**Numerator Df**	***P*-value**
Crop (C)	84.963	3	< 2.2e−16^***^
HG type (H)	1.2603	2	0.532513
Inoculation (I)	20.9222	1	4.78e−06^***^
C × H	0.648	6	0.995546
C × I	12.5867	3	0.005621^**^
H × I	0.8214	2	0.663195
C × H × I	3.7566	6	0.709577

** and ^***^represent significance of χ^2^-test at 0.01, and 0.001, respectively.

Df, degrees of freedom; HG, *Heterodera glycines*; RF, reproductive factor.

**Table 5:** Reproductive factor for crop treatment.

Crop	RF (P_f_/P_i_) at treatment level of	

	2,000 eggs 100/cm^3^	20,000 eggs 100/cm^3^
MN103	1.00 a	0.29 a
MN106	1.13 a	0.33 a
MN108	0.81 a	0.38 a
Susceptible soybean ‘Sturdy’	4.80 b	2.04 b

The values in the same column followed by different letters denote significant differences at the P < 0.05 level using Tukey–Kramer least-square means.

P_final_, egg density at experiment termination; P_initial_, egg density at inoculation; RF, reproductive factor.

### Plant shoot biomass

[1]Likelihood-ratio χ^2^-test for shoot biomass was performed independently for each crop treatment [(C): MN103, MN106, MN108, and soybean]. The factor inoculation (I) was not significant (Table 6). The average shoot biomass values at each inoculation level are reported in Table 7. The three inoculation levels analyzed revealed that there was no difference in average shoot biomass between inoculation levels within individual crop treatment (*P* > 0.05).

**Table 6:** Likelihood-ratio χ^2^-test for crop treatment biomass as influenced by inoculation ([I]: 0, 2,000, and 20,000 eggs/100 cm^3^ soil).

**Crop**	**χ^2^**	**Numerator Df**	***P*-value**
MN 103	3.704	2	0.1569
MN 106	0.393	2	0.8216
MN 108	0.06	2	0.9705
Soybean ‘Sturdy’	2.58	2	0.2752

Df = degrees of freedom.

**Table 7:** Shoot biomass as determined by inoculation level of pennycress and soybean lines.

**Crop**	**Biomass (g) at treatment level of**		

	**0 eggs/100 cm^3^**	**2,000 eggs/100 cm^3^**	**20,000 eggs/100 cm^3^**
MN103	7.13	8.75	8.49
MN106	8.16	8.70	8.30
MN108	9.04	9.05	9.08
Susceptible soybean ‘Sturdy’	12.41	13.92	12.59

## Discussion

This study confirmed the earlier findings in greenhouse studies that pennycress is an alternative host for SCN (Poromarto *et al*., 2015; Venkatesh *et al*., 2000). However, low RF values for the pennycress genotypes indicate that pennycress is a less suitable host for SCN than soybean. An RF value of “1” indicates that the nematode population density was unchanged from the initial population density (or initial inoculation level in this experiment), and an RF<1 indicates that the population density decreased. The average RF value for the pennycress genotypes was <1 at both inoculation levels. The data suggest that the pennycress genotypes showed either no difference or reduced final SCN population density compared to the initial inoculation level. The mechanism for the potential reduction in nematode development on pennycress is unknown, but SCN development is lower for pennycress than on susceptible soybean. Even with the low RF values for pennycress, the crop may maintain SCN population levels in a rotation where the main commodity crop is a host to SCN, such as the corn–soybean relay-cropping system. The analysis of shoot biomass revealed that the inoculation level did not affect the shoot biomass in any crop treatments. One possible explanation is the duration of the experiment. In a field experiment at two sites in Missouri and Iowa, it was found that there was not a significant reduction in soybean stem and leaf weight until 70 d after planting (Wang *et al*., 2003). The present greenhouse experiment was terminated after 60 d. It is plausible that there was not enough time for biomass reduction to occur. However, previous greenhouse studies demonstrated that SCN, at the inoculation level of 10,000 eggs/100 cm^3^ soil, caused significant damage to soybean within the growth period of 65 d after planting. Another possible reason for the lack of SCN effect on soybean and pennycress biomass was less SCN development in the current study than in previous studies (Sun *et al*., 2022).

The limitation of this type of greenhouse evaluation is that temperatures and moisture conditions do not accurately reflect soil and moisture conditions in the soil. As a winter annual, pennycress is present in the cropping rotation when soil temperatures are falling in autumn and warming in spring. It is known that soil temperature plays a key role in the hatching and development of SCN. In both experiments, the temperature was maintained at 28°C, facilitating rapid SCN development. The optimal temperature for SCN development was reported to be 25°C (Alston and Schmitt, 1988). At this temperature, it takes 21 d to–30 d for a full life cycle to be completed (Lauritis *et al*., 1983). SCN development is known to cease when soil temperatures decrease to <10°C, and development to the first-stage juvenile does not occur until the soil temperature increases to 10°C in the spring of the next year (Alston and Schmitt, 1988). The dates of warming and cooling of the soil vary by region, but at the UMN Southern Research and Outreach Center (SROC), the 10-cm soil temperature is <10°C from approximately 15 October to 1 May (Cubins *et al*., 2019; UMN-SROC, 2019). Pennycress yields are optimized when planted from 24 August through 18 September in Minnesota (Dose *et al*., 2017). Depending on the germination rates following pennycress planting, subsequent plant development, and weather conditions, SCN may complete a life cycle in the fall on the established pennycress roots. Development of SCN can also take place during the spring of the year, as the snow melts and the pennycress plants begin to develop and bolt to maturity. Pennycress is harvested from late June through early July, and the soil temperatures rises above 10°C at or near 1 May in Southern Minnesota (Cubins *et al*., 2019; UMN-SROC, 2019). There is a period of up to 60 d when pennycress is in the rotation during which temperatures are suitable for SCN development. Therefore, during spring and early summer, one or more life cycles of SCN reproduction may occur before pennycress senescence and harvest. In the case of pennycress integrated into a rotation with relay-cropped or double-cropped soybean, a “green bridge effect” could result. The “green bridge effect” occurs when one plant facilitates pathogen survival between crop phases and results in greater pest pressure for the soybean crop (Belder *et al*., 2008; Mahoney *et al*., 2016). Although specific research on SCN development on pennycress in the field is lacking, this effect has been observed in other winter annual weeds such as henbit and purple deadnettle. In research completed in production field surveys in Indiana, SCN female development was observed on henbit and purple deadnettle roots in the fall and the spring (Creech *et al*., 2005).

In summary, this study indicates that pennycress is an alternative host for SCN, although there was lower SCN development on pennycress than on SCN-susceptible soybean. Pennycress did not affect the SCN final population density at the low inoculation level and reduced it at the high inoculation level, while susceptible soybean increased the final SCN population density at both levels. Including pennycress as a winter annual cover crop in rotations with soybean can potentially affect SCN density. However, temperature, moisture, and other factors likely play a key role in SCN development under field conditions. This study also demonstrated that SCN, even at high initial population density, had no detectable effect on pennycress or soybean biomass after 60 d. Further studies are needed to determine SCN population development in pennycress under field conditions and the effect of SCN on soybean and pennycress production.
